# An Updated Review of Salivary pH Effects on Polymethyl Methacrylate (PMMA)-Based Removable Dental Prostheses

**DOI:** 10.3390/polym14163387

**Published:** 2022-08-19

**Authors:** Faris A. Alshahrani, Fatemah AlToraibily, Maryam Alzaid, Amr A. Mahrous, Maram A. Al Ghamdi, Mohammed M. Gad

**Affiliations:** 1 Department of Substitutive Dental Sciences, College of Dentistry, Imam Abdulrahman Bin Faisal University, P.O. Box 1982, Dammam 31441, Saudi Arabia; 2 College of Dentistry, Imam Abdulrahman Bin Faisal University, P.O. Box 1982, Dammam 31441, Saudi Arabia

**Keywords:** complete denture, biological and physical properties, polymethylmethacrylate, salivary pH

## Abstract

Salivary pH is a neglected factor that may affect the performance of removable dental prostheses (RDP). This study aimed to review literature in reference to the role of salivary pH on the performance of RDP and materials used for their fabrication. From January 1990 until December 2021, a search was done on PubMed, Scopus, and Web of Science databases using removable dental prostheses, salivary pH, PMMA, Denture base, and physical properties as keywords. Articles that met the inclusion criteria (full-length articles have investigated the effect of salivary pH on RDP materials in vitro and in vivo) were included. Out of 433 articles, 8 articles that met the inclusion criteria were included. All studies used artificial saliva with different salivary pH ranging between 3 and 14. Two articles investigated the role of salivary pH on the cytotoxicity of denture base resins and soft liner. One article studied the durability and retention of attachments, one article analyzed the performance of PEEK materials, one article researched the fatigue resistance of a denture base, one article investigated the corrosion of RPD framework cast and milled Co–Cr, one article studied the strength and clasp retention and deformation of acetal and PEEK materials, and one evaluated changes in mass and surface morphology of CAD–CAM fiber-reinforced composites for the prosthetic framework. Different salivary pH affected all included materials in this review except PEEK materials. The most adverse effect was reported with alkaline and acidic; however, the acidic showed the most deterioration effect. Salivary pH has a role in the selection of material used for RDP fabrication.

## 1. Introduction

With aging, the loss of teeth happens due to trauma, diseases or caries ended by teeth extraction if improperly dentally managed and treated. Total or partial loss of teeth affects function, esthetics, and finally, physiological disturbances [1]. These conditions necessitate the replacement of missing teeth to restore function, esthetics and to regain all defects possible with the optimum prosthetic management [2]. For partially edentulous and completely edentulous patients, removable partial dentures (RPDs) and complete dentures (CDs) have been the optimum treatment options, respectively [3].

Polymethylmethacrylate (PMMA) is the material used for denture base fabrication for CDs and is combined with a cast metal framework for RPDs [4]. Several polymerization processes for the PMMA are used, each of which has its advantages and disadvantages, and accordingly its preference in different procedures in the clinical practice [5]. PMMA has many advantages that have made it suitable to be used up to now, such as esthetics, and it is light in weight, easy to fabricate, easy to repair, and affordable [6]. However, some disadvantages such as low fracture resistance and poor physical properties in different oral fluids as well as allergies were reported and finally affected the clinical performance and denture longevity [7]. To overcome these disadvantages, many attempts have been made through material reinforcements, alternative materials with different compositions, and polymerization techniques [6].

With advanced technology for digital denture fabrications (computer-aided design and computer-aided manufacturing) (CAD–CAM), two methods were used: subtractive (milled) and additive (3D-printing) methods. In the milled method, a pre-polymerized PMMA disc was used to mill the denture base. A milled denture base has many advantages related to the disc fabrication method in which it is fabricated under high temperatures and pressures, which result in a denture base with high strength and adequate surface properties compared to a conventional fabricated one [8]. Moreover, no polymerization shrinkage and less residual monomer give priority to milled over a conventional denture base [9,10].

For RPDs, recently the framework could be fabricated and milled from Polyetheretherketone (PEEK) materials as an alternative to a Co–Cr casted framework [11]. PEEK is a thermoplastic aromatic polymer with high mechanical performance and biocompatibility [11,12]. Moreover, its own semicrystalline structures make it stable under different aging situations and chemically stable, in addition to its ability to resist radiation and sterilization damage [10]. The advantages of PEEK make it appropriate for the RPD framework, including highly esthetic clasps [11,12,13], and could be used in case of allergy to Co–Cr [13,14].

On the level of digital technology and new resin for denture base fabrication, photo-polymerized fluid resins were suggested with different 3D printing systems. The 3D-printed resins have considerable interest and receive more attention and focus from most researchers [15]. In this technology, the denture base was built layer-in-layer from photo-polymerized fluid resins. However, the performance of 3D-printed resins is still low compared to milled and conventional resins [16]. Moreover, the strength and surface properties of 3D-printed resins are obviously affected after thermal cycling [17]. On the level of water sorption and solubility, 3D-printed resins showed more water sorption and solubility compared to conventional ones [18].

For ill-fitted dentures with underneath oral tissue inflammation, refitting using a resilient soft liner was recommended as an optional treatment [19]. In addition to some cases in which patients’ comfort was indicated, such as atrophic ridge management, refitting was also recommended for patients with bruxism, single dentures, a patient with salivary hypofunctions, and xerostomia [20,21]. These resilient materials act as shock absorbents and distribute the stress to supporting tissues due to their viscoelastic properties [22]. Relined dentures, such as a denture, are in contact with oral fluids, which means that soft reline properties may deteriorate [23]. These deteriorations compromised the biological, mechanical, and physical properties of a relined denture base and these deteriorations were time-dependent [24].

Meanwhile, for patients wearing CDs or RPDs, these prostheses are floating in oral fluids and are subjected to other fluids and components with dietary daily intake [25]. Oral fluids compromised different components such as enzymes, proteins, and polysaccharides. Hence, the direct contact between removable prostheses, biodegradation of resin-based restorations, and corrosions of meta-based restorations were expected [26]. The degradation of polymers is mainly attributed to two mechanisms: hydrolysis and salivary enzymatic reactions that resulted in chemical degradation of resin-based restorative material [27]. Therefore, it is essential to select the most appropriate denture base material and the ability to withstand various oral conditions, chewing forces, and thermal and chemical dietary changes in terms of saliva constituents and salivary pH.

Resin-based restorations have an affinity for water uptake, which is considered the main factor contributing to polymer degradation [27]. It was reported that absorbed water resulted in discoloration [28]. The absorbed water diffuses and penetrates the spaces between polymeric chains and forcing them apart causes three-dimensional expansion [18,27]. A direct relation between the amount of absorbed water and the adverse effect of physical properties was reported [27]. For example, as water uptake increases, the dimensional change increases. In terms of mechanical behavior, the absorbed act as a plasticizer that negatively affects the strength of resin-based restorations, and decreases strength, fatigue limit, and surface hardness [17].

Saliva is the first natural biologically produced fluid coming into direct contact with artificially produced dental restoration [29]. Saliva is a clear fluid glandular secretion of the salivary glands that is constantly excreted and poured into the oral cavity via secretory duct openings [30]. Saliva serves many functions, such as keeping the mouth moist and comfortable, and helping in chewing, tasting, and swallowing. In addition, it has proteins that protect the teeth and gingiva. Therefore, its composition, amount, and pH could have an effect on the physical properties of the introduced restoration. Saliva is composed of proteins, enzymes, mucin, proteins, urea, ammonia, and electrolytes such as calcium, sodium, potassium, magnesium, phosphate, and bicarbonate [29]. However, the major component is water, which represents 99% of saliva [29]. The components of saliva interact functionally and play different roles with functions. The bicarbonate, urea, and phosphatase are responsible for buffering the capacity of saliva [29]. Salivary pH normally ranges between 6 and 7. Salivary pH could range from 5.3 in case of low flow to 7.8 in highest flow [29]. Salivary pH can be affected by several factors, including the consumption of different types of food and beverage such as sugar, orange juice, and pastries [30].

Some medical conditions can also increase the acidity of the saliva, such as gastroesophageal reflux, Sjögren’s syndrome, and chemotherapy. Gastroesophageal Reflux Disease (GERD) is an accustomed affection, with approximately 50% of all adults reporting reflux symptoms at some time during their lives [31]. In contrast, the alkalinity of the saliva can be increased due to some types of food or diseases such as problems in the liver and digestive functions including pancreas secretions and enzyme production [30].

The continuous interaction between saliva and removable prostheses necessitates evaluating the effect of salivary pH on mechanical and surface properties of denture base resins, denture lining materials, and RPD framework materials. This is significant, since these materials are subjected to different salivary pH levels on a daily basis, either due to consumption of certain types of acidic or alkaline beverage or diseases [30]. The purpose of this systematic review is to evaluate the effect of different salivary pH on the properties of removable dental prostheses (RDP).

## 2. Materials and Methods

**Figure 1:**
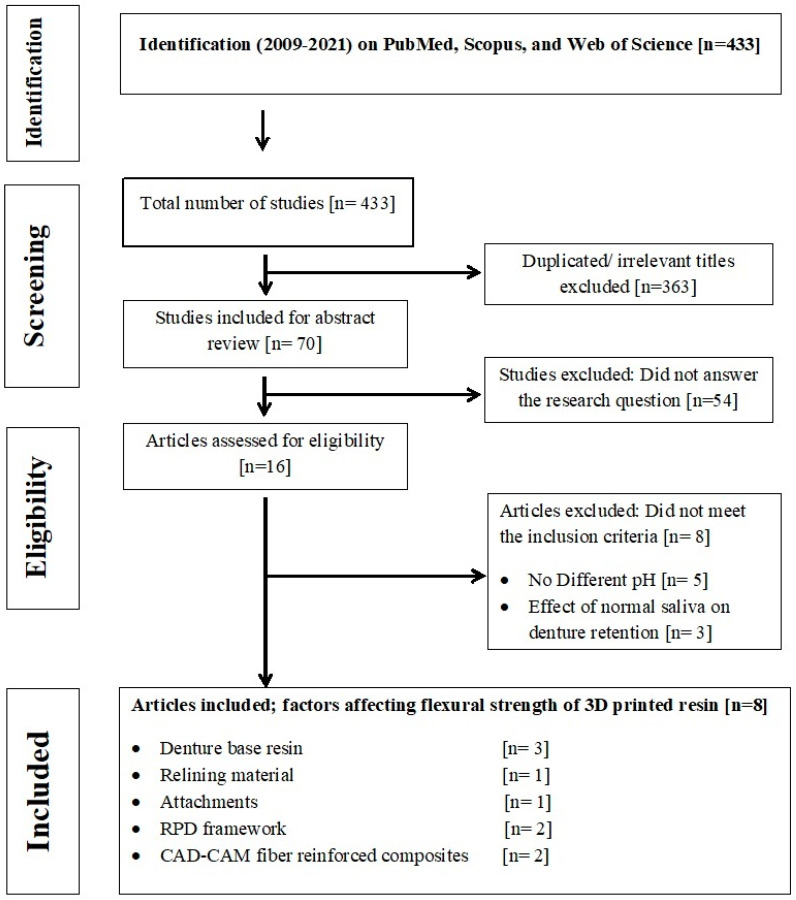
Flow chart of study selection.

The focused study question was: Does salivary pH affect the mechanical and surface properties of RDP? Published articles included in this review reported the original study results that assessed the effect of salivary pH on the properties of materials used for RDP fabrications. A PRISMA (Preferred Reporting Items for Systematic Reviews and Meta-Analysis) guideline was followed to conduct this review (Figure 1) [32].

### 2.1. Eligibility Criteria

Titles inspections of peer-reviewed related published articles were done by two investigators (M.A., F.A.). Exclusions were made for articles that did not measure the effects of salivary pH on denture base materials. In the case of the article, titles were not informative to guide their relevance; abstracts were inspected to verify whether the articles qualified for the study. The included studies met the inclusion criteria: full-length original articles, in vitro and in vivo studies, evaluated the effect of salivary pH on denture base properties, and English-language published.

### 2.2. Search Strategy

This systematic review was performed by searching through PubMed and Scopus, and Web of Science databases to include eligible articles published from January 1990 to December 2021. The following keywords were included: salivary pH, denture base, acrylic resin, and PMMA. To ensure including all related articles, a manual search was used to include references of the relevant review articles.

### 2.3. Data Management, Screening, and Selection

Data were extracted from included studies and tabulated in an Excel sheet including the following information: authors’ names, publication year, denture base types, saliva (groups and composition), sample size, aging, investigated properties, results/outcomes (Table 1). The authors’ discussion was done in case of missing or unclear data to exclude from results analysis if the data were not mentioned. To prove the agreement between investigators in selected studies, the kappa test was used.

### 2.4. Risk of Bias Assessment

Assessments of the articles’ quality was applied individually by three authors using modified Consolidated Standards of Reporting Trials (CONSORT) guidelines [41,42]. Discussion was made between the authors to resolve any confliction. After evaluating the individual article, the parameters were expressed as yes or no (Table 2). Assessment of risk of bias was made according to Joanna Briggs Institute (JBI) [43]. The methodology qualities of selected studies were analyzed using Critical Appraisal Checklist for Quasi-experimental Studies (CACQS—nonrandomized experimental studies). Each study was analyzed independently using nine questions, with options of “yes” “no” “it is not clear” or “not applicable”. The relevant data were collected from studies by two researchers (M.A., F.A.) and then verified by two researchers (F.A.A., M.M.G.) This analysis tabulated data (Table 2) was performed by two investigators (F.A.A., M.M.G.).

Joanna Briggs Institute (JBI) Critical Appraisal Checklist for nonrandomized experimental studies) was used to assess the risk of bias and analyzes the methodological quality of included studies. JBI tools and verification software module were used for each study to be analyzed individually by using nine questions as displayed in Table 2, with options of “yes” “no” “it is not clear” or “not applicable” according to study characteristics. This procedure was done by investigators followed by the calculation of all responses. This analysis was performed by two examiners, and subsequently, the sum of the responses from all studies was calculated [43].

### 2.5. Data Analysis

A descriptive data analysis was applied because of the discrepancies between included studies in terms of prostheses type and methodology (different types of denture base materials, saliva composition, aging effect, and tested properties).

## 3. Results

### 3.1. Data Selection

According to Figure 1, 433 articles were found in PubMed, Scopus, and Web of Science Databases. Duplicated articles (363) and irrelevant articles that didn’t answer the study question (54) were excluded. The eligible articles (16) were subjected to a complete article review to focus on the research study question and met the inclusion criteria. Finally, eight articles [33,34,35,36,37,38,39,40] were included for data extraction (Table 1) and conducting this review. Regarding the kappa test, a high agreement level between investigators was reported (K = 0.89).

### 3.2. Risk of Bias

Table 2 shows the risk of bias in included studies. Hence, “yes” was the answer for most questions, and low risk of bias was reported, which increased the reliability of included studies.

### 3.3. Data Analysis

All studies used artificial saliva with different compositions and a wide range of salivary pH between 3 and 14. The range between 4.3 and 8.3 was more prevalent in most of the studies. The storage and immersion days were 10, 21, and 30 with a recommendation for long immersion duration. Two articles investigated the role of salivary pH on cytotoxicity; one on denture base resin and the second study on soft liner [33,34]. One article evaluated the durability and retention of attachments [35]. One article investigated the performance of PEEK materials [36]. One article evaluated the fatigue resistance of denture base [37]. One article evaluated the corrosion of RPD framework cast and milled Co–Cr [38]. One article evaluated the strength and clasp retention and deformation of acetal and PEEK materials [39]. One article evaluated changes in mass and surface morphology of CAD–CAM fiber-reinforced composites dental materials [40].

The most adverse effect was reported as alkaline and acidic. However, the acidic showed the most deterioration effect. High and low pH increases chemotoxic actions of denture base materials and soft liners. Moreover, the acidic medium has a negative effect on durability and retention. Regarding mechanical behavior, both acidic and alkaline affected the flexural strength, hardness, and elastic modulus of denture base resins and the fatigue resistance, retention, and clasp deformations. More effect was related to the acidic medium. In addition, the metal corrosion for Co–Cr cast or milled metal framework fabricated with an acidic medium was reported. The effect of salivary pH on the surface of composite materials used for the prosthetic framework was found to be less when compared to metal alloys. In between materials, PEEK showed no or fewer effects if present, with different salivary pH.

## 4. Discussion

The normal presence of saliva and denture in the oral cavity refers to correlated effects of the denture on salivary flow and constituents, and the effect of saliva on denture retention and the properties of RDP materials. Denture base materials are subjected in the oral cavity to different conditions such as changes in salivary pH, flow, and temperature. Acidity has a chemical reaction with acrylic resin. It fills the gaps between the polymer chains, which leads to separation in the polymer chains [44], producing unstable polymer chain bonding and causing disruption in the chemical bond. Decreased salivary pH values in the oral environment changes the oral fluids to acidic effects on the characteristics, properties, and behavior of dental materials. The study question of this review focused on the effect of different salivary pH on the properties of RDP. RDP are prone to several environmental factors affecting their properties. These factors include humidity, changes in temperature, and saliva. Salivary pH changes (varying states of alkalinity to acidity) have been an interesting subject in the field of removable dental prostheses [26].

Normal salivary pH is 6–7 slightly acidic; however, it can range from 5.3 to 7.8 based on the oral environment [29]. Salivary pH changes with dietary foods and with the presence of some diseases [29,44]. The instability of salivary pH could be due to the response of human saliva to numerous factors throughout the day resulting in increased or decreased salivary pH [45]. A lower pH was reported with the consumption of orange juice, candy, smoking or an alkaline, on eating amaranth or in case of increased secretion of pancreatic juice in addition to the amount of sugar consumed and carbohydrate fermentation [39]. GERD is the most predominant digestive disease today; it has been reported that 50% of adults have reflux symptoms during their lives [31]. GERD can cause a reduction in oral pH [45]. Moreover, patients with xerostomia and hyposalivation are more prone to have low salivary pH; this is due to the diminished buffering effect of saliva when it is produced in a low amount. The imbalance in buffering effect resulted in more microorganisms in the oral cavity, which subsequently increases the medium acidity even more [30]. Sjögren’s syndrome and chemotherapy also resulted in acidic pH [46]. Saliva pH could have high alkalinity due to consumption of some foods or a disease such as digestive, liver, and lymphatic system dysfunctions [47].

Wearing dental prostheses can be complicated with Prosthetic Stomatitis due to increased growth of fungus. Studies found that low salivary pH can cause a decrease in micro-hardness as a result of the degradation of acrylic; moreover, it can lead to a more residual monomer release and a decrease in fracture resistance. Hence, dental prostheses subject to low salivary pH levels are more likely to be fragile and susceptible to fracture [37,47]. Dental materials are continuously subjected to different oral environments and to the oral microbiome metabolism. This can decrease the lifetime of prosthetic restorations used by patients. Moreover, the dental prosthesis can be affected by the aging process while in use due to different clinical conditions, for instance changes in salivary pH, flow, and temperature [48]. Therefore, the most suitable material for prosthesis fabrication that could withstand all conditions is required. The review question answer is yes, where materials used for RDP fabrication were affected by different pH. The results reveal the daily effect on patients’ acrylic prostheses because there are many reasons for decreased salivary pH [45].

Acrylic resin can be degraded by saliva, chewing, and diet. Resin molecules have polar properties and hence acrylic resin absorbs water when immersed and leads to the diffusion of free monomers and other products. While the denture is inserted, denture plaque is formed. Saliva and denture are acidified by fermentative and dietary acids, resulting in the exposure of denture surfaces to acidic environments [33]. The components’ leachability of the resin-based polymer was reported in normal conditions with limited effects on the oral tissues. This mild effect increased as salivary pH changed to either acidic or alkaline, resulting in increasing leachability, and subsequently the adverse effect in terms of chemotoxics increased [27,29,33,34]. It was reported that material first has hydrolysis in saliva and then leaches out due to instability in the saliva rather than pH-dependency in leachability itself [33]. One of the biggest drawbacks of denture base resin is the residual monomer, which is reported as a tissue irritant [27]. Therefore, the selection of material with less residual monomer is recommended to be used with different salivary pH such as the newly suggested pre-polymerized CAD–CAM blocks [49]. To confirm this presumption for resin-based polymer, further investigations are required with immersion in artificial saliva with different salivary pH and solutions with diverse acidity.

Denture liners used under the denture are subjected to plaque accumulation, leading to a decrease in salivary pH of less than 7; this is due to plaque bacteria provoking sugars and producing lactic acid [34]. During the use of soft liner materials, they undergo two reactions: the soluble components and the plasticizers are leached out and the water or saliva is absorbed inside the voids [50]. Denture liners may compromise the leach-out ingredients or extrinsic ingredients that may integrate into the material and, consequently, lead to loss of the ethanol and plasticizer or material degradation. These deterioration events can change the overall performance of soft liner materials over time [23]. Song et al. [51] found that the level of cytotoxic ingredients leached from the material will considerably reduce before they pass through the oral mucosa; hence, it will be diluted by saliva. One of the common drawbacks of soft denture liners is water sorption and solubility, and it was found that soft linings exhibited high solubility in artificial saliva [23]. This issue is correlated with the changes in the structure, physical properties [52], and the dimensional stability of the materials that may cause swelling, distortion, change in color, increased *Candida albicans* growth, and reduced bond strength by causing tensions at the liner denture base interface [50].

For removable prostheses treatment, two treatment modalities were reported: temporary or definitive. In case of temporary it is recommended to use a maximum of 6 months or until definitive treatment procedures are completed. In this review, all removable prostheses and related parts investigated in the included studies are under definitive treatment categories [33,34,35,36,37,38,39,40]. Generally, properties of resin-based polymers were adversely affected by salivary pH, especially low pH levels. The surface roughness of heat polymerized material increased when immersed in different salivary pH. This effect may be attributed to the fact that the neutral and acidic pH values can affect the degradation ratios, while in basic pH values there is an elevated level of Hydroxyl ions, causing acceleration in the degradation and leading to high surface roughness [53].

When metal is a part of the RDP, more attention to fluid-related properties must be considered, as the RPDs are in direct contact with the oral fluid. This is presented in a phenomenon called corrosion. For Implant-supported overdenture, the attachments are subjected to different salivary pH. Under an acidic environment, it was found that the attachments exhibit decreases in retention. The attachment durability is affected by both mechanical factors of wear and chemical phenomena such as corrosion, noting that these factors may differ in the oral cavity from patient to patient. Corrosion also can occur in attachment elements of overdenture, affecting durability and retention, variable from person to person [35].

From dental alloys, Co–Cr alloys were selected for dental restorations because they are chemically inert, with low irritations and allergic reactions. Additionally, their corrosion resistance reduces the complications in the section part of the oro-facial system [54,55]. However, corrosion resistance could be reduced due to variation in salivary pH [56]. It was found that in high acidic artificial salivary pH, Co–Cr alloy revealed a poorer corrosion resistance, irrespective of the manufacturing technique used (cast or milled). It was noted that milled Co–Cr alloy had higher corrosion resistance compared to cast Co–Cr alloy. Considering the lowest corrosion rate values of milled Co–Cr alloy in an acidic environment, we can declare that this type of alloy presents a superior option for the prosthetic treatment of patients suffering from GERD [38].

PEEK is a thermoplastic aromatic polymer that has biocompatibility and higher mechanical properties [12]. Therefore, PEEK material is considered an excellent material for RDP framework fabrications [12]. It was reported that low salivary pH had a noticeable effect on the mechanical properties of the tested resins (PEEK, acetal, and heat-cured polymer composite) followed by high salivary pH after thermal cycles [39]. Acetal clasp tips showed more deformation, and this may be due to the flexibility as stated in a previous study [57]; clasp with greater flexibility has higher deformation when subjected to fatigue aging. Heat-cured polymer composite material showed the lowest mechanical performance among tested resins [39]. This is mainly attributed to the high water sorption, which results in the expansion of PMMA in addition [36] to the ability of solvent particles to penetrate into the PMMA matrix and disrupt the intermolecular bonding between polymer networks [39]. However, PEEK resin showed no significant change with different salivary pH [39]. The same behavior was reported with novel composite biomaterials that were suggested to be used as substitute for metal alloys used for prosthetic frameworks for oral rehabilitation of patients having GERD [40].

The risk of bias of included studies was low to moderate, so the finding of this review should be interpreted with caution. This is also related to the high variations in the testing methodology, the material used, the nature of the test, and the measuring methods. Clinically, the behavior of materials used for RDP fabrication with different salivary pH must be considered an important factor for material selection. CAD–CAM materials (PEEK and milled Co–Cr) showed less to no effect with different salivary pH; however, further investigations of salivary pH effect on different CAD/CAM materials used for RDP fabricated are recommended. In some cases, with border pH such as GERD, Sjögren’s syndrome, Xerostomia, and/or drug-induced xerostomia and hyposalivation, patients under chemotherapy required materials with high corrosion resistance and stability under different salivary pH. The material that could withstand the deferent environment has the selection priority. Attention to PEEK performance in further investigations would be of interest confirming its performance for RDP fabrication.

The low number of included in vitro studies is considered one limitation of this review. Wide variations in pH values of artificial saliva between included studies as well as the immersion times are considered limitations to conduct systematic review and to end with a proper and clear conclusion. Therefore, a further investigation with ranges close to human saliva is required as well as including more articles conducting a systematic review.

## 5. Conclusions

Because RDP are in constant contact with oral fluids, the effect of these fluids on their performances is recommended. Although various studies investigated the performance of RDP material, a low number of studies investigated the effect of salivary pH. Salivary pH affected the biocompatibility and physical properties of materials used for RDP fabrication. The oral environment with different salivary pH should be considered during the selection of materials used for RDP fabrication. Due to a lack of information about salivary pH effects on CAD–CAM materials, further investigations are required.

## Figures and Tables

**Table 1:** Details of included studies.

Author/Year/ Type of Study	Restorations/Specimen Dimensions	Saliva/Type		Sample Size	Aging Effect	Tested Properties	Results and Outcome
		pH	Composition				
Koda T et al., 1990 [33]	Auto-polymerized Heat-polymerized microwave-polymerized/ Resin disks (thickness of 2.0 × 8.5 mm	pH 4 pH 5.0 pH 6.0 pH 6.8	0.3 mM CaS0_4_; 1.0 mM NaCl; 0.7 mM KC1; 0.4 mM KH2P0_4_ 0.4 mM Na2HP0_4_	(*n* = 10)	10 days immersion	Leachability of MMA, methacrylic acid (M), benzoic acid (BA), and methyl acrylate (MA),	The leachability of MMA, M, BA, and MA increased in more acidic and less acidic pH and this mainly due hydrolysis of MMA which resulted in more chemotoxic actions of denture base material
Akay C et al., 2017 [34]	denture lining materials/ disk 5-mm x 2-mm thickness	pH 4 pH 7 pH 14	4.1 mM KH2PO_4_, 4.0 mM Na2 HPO_4_, 24.8 mM KHCO_3_, 16.5 mM NaCl, 0.25 mM CaCl_2_.	(*n* = 96) (*n* = 12)	21 days immersion	Cytotoxicity	The cytotoxicity of soft liners increases with storage in different salivary pH.
Silva et al., 2015 [35]	4 types of attachments	pH 4 pH 7	KC1;18.5% 6.5 mL NaCl 20% 8.6mL CaCl2•2H_2_O 10% 8 mL Sorbitol 50% 48 mL Carboxymethylcellulose 20 g Citric acid 10 g Nipagin 1.6 g Nipasol 0.4 g Water 1950 mL	(*n* = 4)	5400 insertion/removal cycles were simulated (5 years)	durability and retention	Different salivary pH adversely affects the retention and the most negative effect on the attachment retention was recorded with more acidic pH.
Gao et al., 2015 [36]	polyetheretherketone (PEEK) and a carbon fiber-reinforced PEEK (CFR-PEEK) with 30% short carbon fibers, a dental composite based on Bis-GMA and polymethylmethacrylate (PMMA)/ milled disks of 8 × 10 mm	pH 3 pH 7 pH 10	NaCl 125.6 KCl 963.9 KSCN 189.2 KH2PO_4_ 654.5 Urea 200.0 NaSO4·10H2O 763.2 NH4Cl 178.0 CaCl_2_·2H_2_O 227.8 NaHCO3 630.8	(*n* = 40/material) (*n* = 10/pH)	30 days immersion	elastic moduli, nanohardness, viscoelasticity, and friction performance	no significant changes in PEEK after immersion in different salivary pH
Sa et al., 2019 [37]	prosthesis bases	pH 4 pH 7	0.4 g/L NaCl, 0.4 g/L KCl, 0.795 g/L CaCl2.2H_2_O, 0.005 g/L Na2S.9H_2_O, 0.69 g/L NaH_2_PO_4_.2HSO, 1 g/L urea	(*n* = 5)	30 days immersion	fatigue resistance	the fracture resistance of denture base resins was decreases with results at low pH environment
Bechir et al., 2021 [38]	Co-Cr alloy, casted or milled/ discs with a diameter of 15 mm and a thickness of 5 mm.	pH 3 pH 5.7 pH 7.6	Na2HPO4 0.19 NaCl 0.7 KSCN 0.33 KH2PO_4_ 0.26 NaHCO_3_ 1.5 Ureea 1.3	NS	NS	corrosion behavior of two commercial Co-Cr dental alloys manufactured by casting and by milling	Co-Cr alloys (cast and milled) have poor corrosion resistance when immersed in artificial saliva with acidic salivary pH. However, the corrosion behavior of milled one was better, making this alloy a better option for GERD patients
Fathy et al., 2021 [39]	denture base and clasp construction/ Two Aker clasps materials (acetal and PEEK) 65× 10 ×2.5 mm 92 disk-shaped (10 × 1 mm)	pH 5.8 pH 7.2 pH 8.3	NaCl 0.70, Na_2_PO_4_ 0.26, KSCN 0.33, KH_2_PO_4_ 0.20	(*n* = 10)	NS	flexural strength, hardness, clasp retention and deformation	At acidic and alkaline pH and combining thermal aging, the flexural strength and surface microhardness of acetal, as well as its clasp retention and deformation, Meanwhile, PEEK clasps were not significantly affected.
Bechir et al., 2021 [40]	restorative materials for prosthetic oral rehabilitation Two CAD/CAM Fiber-Reinforced Composite Dental/ 15 mm × 5 mm	pH 3 pH 5.7 pH 7.6	Na_2_HPO_4_ 0.19 g NaCl 0.70 g KSCN 0.33 g KH_2_PO_4_ 0.26 g NaHCO_3_ 1.50 g Urea 1.30 g	NS	21 days	changes in mass or surface morphology	Novel composite biomaterials showed a stable surface when in contact with different salivary pH and can be used to fabricate prosthetic frameworks in GERD patients

**Table 2:** Risk of bias analysis of included studies.

Questions—JBI Critical Appraisal Checklist		Revisor 1				Revisor 1			
		Yes	No	Unclear	NA	Yes	No	Unclear	NA
1	Is it clear in the study what is the ‘cause’ and what is the ‘effect’ (there is no confusion about which variable comes first)?	8				8			
2	Were the participants included in any comparisons similar?	8				8			
3	Were the participants included in any comparisons receiving similar treatment/care, other than the exposure or intervention of interest?	5		3		8			
4	Was there a control group?	8				8			
5	Were there multiple measurements of the outcome both pre and post the intervention/exposure?	6		2		8			
6	Was follow up complete and if not, were differences between groups in terms of their follow up adequately described and analyzed?				8				8
7	Were the outcomes of participants included in any comparisons measured in the same way?	8			7			1	
8	Were outcomes measured in a reliable way?	6		2	8				
9	Was appropriate statistical analysis used?	8			8				

## Data Availability

All data included in the review.

## References

[1] Gupta A., Felton D.A., Jemt T., Koka S. (2019). Rehabilitation of Edentulism and Mortality: A Systematic Review. J. Prosthodont..

[2] Muller F., Schimmel M. (2010). Tooth loss and dental prostheses in the oldest old. Eur. Geriatr. Med..

[3] Friel T., Waia S. (2020). Removable Partial Dentures for Older Adults. Prim. Dent. J..

[4] Zafar M.S. (2020). Prosthodontic Applications of Polymethyl Methacrylate (PMMA): An Update. Polymers.

[5] Khan A.A., Fareed M.A., Alshehri A.H., Aldegheishem A., Alharthi R., Saadaldin S.A., Zafar M.S. (2022). Mechanical Properties of the Modified Denture Base Materials and Polymerization Methods: A Systematic Review. Int. J. Mol. Sci..

[6] Gad M.M., Fouda S.M., Al-Harbi F.A., Näpänkangas R., Raustia A. (2017). PMMA denture base material enhancement: A review of fiber, filler, and nanofiller addition. Int. J. Nanomed..

[7] Abualsaud R., Gad M.M. (2022). Flexural Strength of CAD/CAM Denture Base Materials: Systematic Review and Meta-analysis of In-vitro Studies. J. Int. Soc. Prev. Community Dent..

[8] Steinmassl O., Dumfahrt H., Grunert I., Steinmassl P.A. (2018). CAD/CAM produces dentures with improved fit. Clin. Oral Investig..

[9] Baba N.Z., Goodacre B.J., Goodacre C.J., Müller F., Wagner S. (2021). CAD/CAM Complete Denture Systems and Physical Properties: A Review of the Literature. J. Prosthodont..

[10] Prpić V., Schauperl Z., Ćatić A., Dulčić N., Čimić S. (2020). Comparison of Mechanical Properties of 3D-Printed, CAD/CAM, and Conventional Denture Base Materials. J. Prosthodont..

[11] Stawarczyk B., Beuer F., Wimmer T., Jahn D., Sener B., Roos M., Schmidlin P.R. (2013). Polyetheretherketone-a suitable material for fixed dental prostheses?. J. Biomed. Mater. Res. B Appl. Biomater..

[12] Schwitalla A.D., Spintig T., Kallage I., Müller W.D. (2015). Flexural behavior of PEEK materials for dental application. Dent. Mater..

[13] Chen X., Mao B., Zhu Z., Yu J., Lu Y., Zhang Q., Yue L., Yu H. (2019). A three-dimensional finite element analysis of mechanical function for 4 removable partial denture designs with 3 framework materials: CoCr, Ti-6Al-4V alloy and PEEK. Sci. Rep..

[14] Lekha K., Savitha N., Roseline M., Nadiger R.K. (2012). Acetal resin as an esthetic claspmaterial. J. Interdiscip. Dent..

[15] Alghazzawi T.F. (2016). Advancements in CAD/CAM technology: Options for practical implementation. J. Prosthodont. Res..

[16] Anadioti E., Musharbash L., Blatz M.B., Papavasiliou G., Kamposiora P. (2020). 3D printed complete removable dental prostheses: A narrative review. BMC Oral Health.

[17] Gad M.M., Fouda S.M., Abualsaud R., Alshahrani F.A., Al-Thobity A.M., Khan S.Q., Akhtar S., Ateeq I.S., Helal M.A., Al-Harbi F.A. (2021). Strength and Surface Properties of a 3D-Printed Denture Base Polymer. J. Prosthodont..

[18] Gad M.M., Alshehri S.Z., Alhamid S.A., Albarrak A., Khan S.Q., Alshahrani F.A., Alqarawi F.K. (2022). Water Sorption, Solubility, and Translucency of 3D-Printed Denture Base Resins. Dent. J..

[19] Nowakowska-Toporowska A., Malecka K., Raszewski Z., Wieckiewicz W. (2019). Changes in hardness of addition-polymerizing silicone-resilient denture liners after storage in artificial saliva. J. Prosthet. Dent..

[20] Hashem M.I. (2015). Advances in soft denture liners: An update. J. Contemp. Dent. Pract..

[21] Usta Kutlu I., Yanikoğlu N.D., Kul E., Duymuş Z.Y., Sağsöz N.P. (2016). Effect of sealer coating and storage methods on the surface roughness of soft liners. J. Prosthet. Dent..

[22] Landayan J.I.A., Manaloto A.C.F., Lee J.Y., Shin S.W. (2014). Effect of aging on tear strength and cytotoxicity of soft denture lining materials; in vitro. J. Adv. Prosthodont..

[23] Yanikoglu N.D., Duymuş Z.Y. (2004). Comparative study of water sorption and solubility of soft lining materials in the different solutions. Dent. Mater. J..

[24] Takahashi Y., Hamanaka I., Shimizu H. (2013). Flexural properties of denture base resins subjected to long-term water immersion. Acta Odontol. Scand..

[25] Gale M.S., Darvell B.W. (1999). Thermal cycling procedures for laboratory testing of dental restorations. J. Dent..

[26] Muddugangadhar B.C., Sangur R., Rudraprasad I.V., Nandeeshwar D.B., Kumar B.H. (2015). A clinical study to compare between resting and stimulated whole salivary flow rate and pH before and after complete denture placement in different age groups. Indian Prosthodont. Soc..

[27] Bettencourt A.F., Neves C.B., de Almeida M.S., Pinheiro L.M., Oliveira S.A., Lopes L.P., Castro M.F. (2010). Biodegradation of acrylic based resins: A review. Dent. Mater..

[28] Polychronakis N., Polyzois G., Lagouvardos P., Andreopoulos A., Ngo H.C. (2018). Long-term microwaving of denture base materials: Effects on dimensional, color and translucency stability. J. Appl. Oral Sci..

[29] Humphrey S.P., Williamson R.T. (2001). A review of saliva: Normal composition, flow, and function. J. Prosthet. Dent..

[30] Hussein Y.A., Al-Ameer S.S. (2012). The influence of different pH of saliva and thermal cycling on the adaptation of different denture base materials. J. Bagh. Coll. Dent..

[31] Antunes C., Aleem A., Curtis S.A. (2018). Gastroesophageal Reflux Disease (GERD). Mo. Med..

[32] Shamseer L., Moher D., Clarke M., Ghersi D., Liberati A., Petticrew M., Shekelle P., Stewart L.A., the PRISMA-P Group (2015). Preferred reporting items for systematic review and meta-analysis protocols (PRISMA-P)2015: Elaboration and explanation. BMJ.

[33] Koda T., Tsuchiya H., Yamauchi M., Ohtani S., Takagi N., Kawano J. (1990). Leachability of denture-base acrylic resins in artificial saliva. Dent. Mater..

[34] Akay C., Tanış M.Ç., Sevim H. (2017). Effect of artificial saliva with different pH levels on the cytotoxicity of soft denture lining materials. Int. J. Artif. Organs..

[35] Silva A.S., Aroso C., Ustrell R., Braga A.C., Mendes J.M., Escuin T. (2015). The influence of saliva pH value on the retention and durability of bar-clip attachments. J. Adv. Prosthodont..

[36] Gao S., Gao S., Xu B., Yu H. (2015). Effects of Different pH-Values on the Nanomechanical Surface Properties of PEEK and CFR-PEEK Compared to Dental Resin-Based Materials. Materials.

[37] de Sá J., Vieira F., Aroso C.M., Cardoso M., Mendes J.M., Silva A.S. (2020). The Influence of Saliva pH on the Fracture Resistance of Three Complete Denture Base Acrylic Resins. Int. J. Dent..

[38] Bechir F., Bataga S.M., Ungureanu E., Vranceanu D.M., Pacurar M., Bechir E.S., Cotrut C.M. (2021). Experimental Study Regarding the Behavior at Different pH of Two Types of Co-Cr Alloys Used for Prosthetic Restorations. Materials.

[39] Fathy S.M., Emera R.M.K., Abdallah R.M. (2021). Surface Microhardness, Flexural Strength, and Clasp Retention and Deformation of Acetal vs Poly-ether-ether Ketone after Combined Thermal Cycling and pH Aging. J. Contemp. Dent. Pract..

[40] Bechir F., Bataga S.M., Tohati A., Ungureanu E., Cotrut C.M., Bechir E.S., Suciu M., Vranceanu D.M. (2021). Evaluation of the Behavior of Two CAD/CAM Fiber-Reinforced Composite Dental Materials by Immersion Tests. Materials.

[41] Bangera M.K., Kotian R.N.R. (2020). Effect of titanium dioxide nanoparticle reinforcement on flexural strength of denture base resin: A systematic review and meta-analysis. Jpn. Dent. Sci. Rev..

[42] Faggion C.M. (2012). Guidelines for reporting pre-clinical in vitro studies on dental materials. J. Evid. Based Dent. Pract..

[43] de Oliveira Limírio J.P.J., Gomes J.M.L., Alves Rezende M.C.R., Lemos C.A.A., Rosa C.D.D.R.D., Pellizzer E.P. Mechanical properties of polymethyl methacrylate as a denture base: Conventional versus CAD-CAM resin—A systematic review and meta-analysis of in vitro studies. J. Prosthet. Dent..

[44] Sofya P.A., Rahmayani L., Purnama R.R. (2017). Effect of soft drink towards heat cured acrylic resin denture base surface roughness. Padjadjaran J. Dent..

[45] Lussi A., Jaeggi T., Zero D. (2004). The role of diet in the aetiology of dental erosion. Caries Res..

[46] Minich D.M., Bland J.S. (2007). Acid-alkaline balance: Role in chronic disease and detoxification. Altern. Ther. Health Med..

[47] Figueiral M.H., Azul A.M., Fonseca P., Pinto E., Branco F.M. (2006). Influence of saliva on prosthetic stomatitis. Rev. Port. Estomatol. Med. Dent. E’Cir. Maxilofac..

[48] Garcia L.F., Roselino L.M., Mundim F.M., Consani S. (2010). The influence of artificial accelerated aging on dimensional stability of acrylic resin submitted to different storage protocols. J. Prosthodont..

[49] Wei X., Pan Y., Wang M., Wang Y., Lin H., Jiang L., Lin D., Cheng H. (2022). Comparative analysis of leaching residual monomer and biological effects of four types of conventional and CAD/CAM dental polymers: An in vitro study. Clin. Oral Investig..

[50] El-Hadary A., Drummond J.L. (2000). Comparative study of water, Solubility, and tensile bond strength of two soft lining materials. Prosthet. Dent..

[51] Song Y.H., Song H.J., Han M.K., Yang H.S., Park Y.J. (2014). Cytotoxicity of soft denture lining materials depending on their component types. Int. J. Prosthodont..

[52] Khan A.A., De Vera M.A., Mohamed B.A., Javed R., Al-Kheraif A.A. (2022). Enhancing the physical properties of acrylic resilient denture liner using graphene oxide nanosheets. J. Vinyl Addit. Technol..

[53] Achim G. (1996). Mechanisms of Polymer Degradation and Erosion. Biomaterials.

[54] Armencia A.O., Hurju I., Tărniceriu C.C., Lese A., Feier R., Scutariu M.M., Balcos C. (2020). The study of roughness and resistance to corrosion of dental alloys in the oral environment. Rom. J. Oral Rehabil..

[55] Kassapidou M., Stenport V.F., Hjalmarsson L., Johansson C.B. (2017). Cobalt-chromium alloys in fixed prosthodontics in Sweden. Acta Biomater. Odontol. Scand..

[56] Soares F.M.D.S., Santana A.I.D.C., dos Santos L.B.F., Gomes P.A.M.C., Monteiro E.D.S., Coimbra M.E.R., Elias C.N. (2019). Influence of oral pH Environment in the Corrosion Resistance of Cr-Co-Mo alloy Used for Dentistry Prosthetic Components. Mater. Res..

[57] Najeeb S., Zafar M.S., Khurshid Z., Siddiqui F. (2016). Applications of polyetheretherketone (PEEK) in oral implantology and prosthodontics. J. Prosthodont. Res..

